# Micro Coriolis Mass Flow Sensor with Large Channel Diameter Realized by HNA Wet Etching

**DOI:** 10.3390/s24247952

**Published:** 2024-12-12

**Authors:** Qihui Yu, Maarten J. S. Bonnema, Mahdieh Yariesbouei, Remco J. Wiegerink, Joost C. Lötters

**Affiliations:** 1 MESA^+^ Institute for Nanotechnology, University of Twente, 7522 NH Enschede, The Netherlandsr.j.wiegerink@utwente.nl (R.J.W.); j.c.lotters@utwente.nl (J.C.L.); 2 Institute for Biomedical Engineering, University and ETH Zürich, 8092 Zürich, Switzerland; 3 Paul Scherrer Institute, 5232 Villigen, Switzerland; 4 Philips Medical Systems International B.V., 5684 PC Best, The Netherlands; mahdieh.yari@gmail.com

**Keywords:** surface channel technology, HNA etching, microfluidic channel, Coriolis mass flow sensor

## Abstract

This paper introduces a Coriolis mass flow and density sensor. The sensor is made using Surface Channel Technology (SCT) but with selective wet etching to create the channels. This method forms suspended microfluidic channels with a larger cross-sectional area. Because of this larger cross-sectional area, the sensor has a much higher flow range, up to 50 $g\;h^{-1}$ (for water) with a pressure drop of 1 bar, compared to the standard SCT-based Coriolis sensor, which is only 1.2 $g\;h^{-1}$. The channel has a semi-elliptical cross-sectional area, measuring 200 micrometers wide and 70 micrometers deep. The channel wall is made of a stack of thin films with a total thickness of 2.5 μm. Water, isopropyl alcohol (IPA), and nitrogen (N_2_) are used to test and evaluate the sensor’s mass flow and density sensing performance.

## 1. Introduction

Accurate flow measurement and control are important in many applications, for example, in gas chromatography [1], biomedical applications [2], micro mixers and reactors [3], and mechanical ventilation [4]. There are many types of flow sensors. Coriolis mass flow sensors stand out because they measure true mass flow by detecting vibrations in a suspended tube structure, independent of the fluid’s properties. This allows for accurate flow measurements even if the fluid type changes, so no recalibration is needed. Additionally, Coriolis sensors can measure fluid density. This works by measuring how the tube’s vibration frequency changes since a different fluid density affects the tube’s mass.

The first silicon micromachined Coriolis mass flow sensor was presented by Enoksson et al., in the late 1990s [5,6]. Since then, several other research groups have been working on miniature Coriolis mass flow sensors [7,8,9,10,11,12]. In 2007, the so-called Surface Channel Technology (SCT) was proposed by Dijkstra et al. [13]. This technology allows the fabrication of suspended channels with a thin silicon-rich silicon nitride (SiRN) tube wall in a single silicon substrate, and a Coriolis mass flow sensor fabricated in SCT was demonstrated by Haneveld et al. [14]. Numerous studies and projects of Coriolis mass flow sensors fabricated with SCT were carried out in the last two decades, focusing on device design and modeling [15], actuation and readout methods, integration [16,17], and the integration of multiple sensors [18]. However, the cross-sectional size of the microfluidic channels fabricated with SCT is limited, usually with a diameter between 40 and 80 μm, and a channel wall thickness between 1.2 and 1.5 μm [19]. The demonstrated Coriolis mass flow sensors fabricated with SCT usually have a flow range of 0–1.2 $g\;h^{-1}$ [15] and a maximum of up to 10 $g\;h^{-1}$ for water measurement [20]. Yariesbouei et al. [9] discussed the diameter-to-wall-thickness ratio and the effect on the performance of the sensor. To further expand the flow range while keeping the advantages of the SCT channel (thin and chemically inert channel wall, easy integration of actuation/readout system, etc.), it is necessary to modify the existing SCT or to investigate new fabrication methods to realize microfluidic channels with a larger cross-sectional area. Therefore, in [21], we proposed to modify the fabrication process, using wet etching by HNA to etch the channel.

In this paper, we present a Coriolis mass flow sensor consisting of a U-shaped tube loop with dimensions of 3.5 mm × 5 mm, a tube width of 200 μm, a depth of 70 μm, and a wall thickness of 2.5 μm, realized by using the HNA etching method, with the results of mass flow and density measurements using water, IPA, and nitrogen. First, in Section 2, the operating principle and the design of the sensor are presented. A detailed description of the fabrication process is given in Section 3. The experimental method is introduced in Section 4, followed by the result and discussion in Section 5.

## 2. Operating Principle and Sensor Design

**Figure 1:**
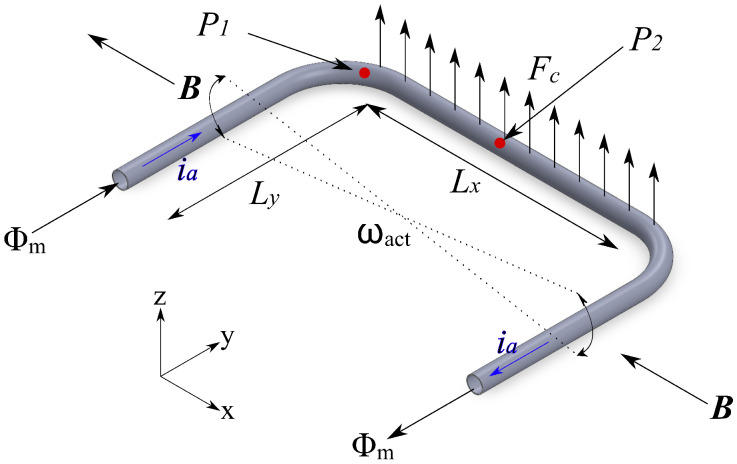
Basic structure and operating principle of the Coriolis mass flow sensor.

A Coriolis mass flow sensor measures mass flow by generating and detecting the Coriolis force, which depends directly on the mass flow. Figure 1 shows the basic structure of the sensor. The tube is actuated to vibrate around the *y* axis in a “twist mode” with an angular velocity $\omega_{act}$. Lorentz force actuation [17] can be used by applying an alternating current $i_{a}$ through the metal tracks on the tube, in the presence of an external magnetic field (***B***). When mass flow $\Phi_{m}$ passes through the tube, it creates a Coriolis force $F_{c}$ perpendicular to the axis of rotation. This can be expressed as [15]

(1) $$\vec{F_{c}}=-2L_{x}\left({\vec{\omega }}_{act}\times {\vec{\Phi }}_{m}\right)$$

Here, *L* is the length of the tube segment shown in Figure 1. This Coriolis force causes the tube to vibrate in a “swing mode”, where the vibration amplitude is directly linked to the mass flow. Therefore, measuring this vibration provides the mass flow value.

### 2.1. Pressure Drop

According to the Hagen–Poiseuille law [22] for a laminar, incompressible Newtonian fluid flow in a channel, the mass flow is proportional to the square of the cross-sectional area with a given pressure drop and channel length. The mass flow can be expressed as

(2) $$\Phi_{m}=\frac{\Delta PA^{2}\rho }{8\pi \mu L}$$

where $\Phi_{m}$ is the mass flow, ΔP is the pressure drop, *A* is the cross-sectional area, ρ is the fluid density, μ is the dynamic viscosity, and *L* is the length of the channel. Moreover, according to Schut et al. [23], for a laminar flow in a channel, there is an additional pressure drop due to the bends and junctions of the channel, and it can be expressed as

(3) $$\Delta P=\frac{1}{2}\rho u^{2}\kappa$$

where ρ is the density of fluid, *u* is the flow velocity, and κ is the loss coefficient. The total pressure drop along the channel can be calculated by summing the pressure drop over the straight sections (Equation (2)) and corners (Equation (3)). It can be expressed as

(4) $$\Delta P_{total}=\frac{\kappa }{2A^{2}\rho }\Phi_{m}^{2}+\frac{8\pi \mu L}{A^{2}\rho }\Phi_{m}$$

### 2.2. Density Measurement

The resonance frequency of the tube changes when filled with different fluid media. This change is due to the change in the total mass of the vibrating structure. Since the inner volume of the tube remains unchanged, the change in resonance frequency is a measure for fluid media density. Yariesbouei et al. [9] derived the the following expression for the resonance frequency $f_{0}$:

(5) $$f_{0}=\frac{1}{2\pi }\sqrt{\frac{K_{0}+\alpha P}{m_{tube}+\rho V_{tube}}}$$

where $K_{0}$ is the effective modal spring constant at atmospheric pressure, *P* and α are the gauge pressure and pressure dependence coefficient, respectively, $m_{tube}$ is the mass of the empty channel, ρ is the density of the fluid in the channel, and $V_{tube}$ is the inner volume of the channel. From Equation (5), one can derive that

(6) $${\left(\frac{1}{f_{0}}\right)}^{2}=\frac{4\pi^{2}V_{tube}}{K_{0}+\alpha P}\rho +\frac{4\pi^{2}m_{tube}}{K_{0}+\alpha P}$$

### 2.3. Coriolis Mass Flow Sensor Modeling

In 2010, Haneveld et al. developed an analytical model for Coriolis mass flow sensors [15] by describing the angular rotation of the two modes (twist and swing) with the following differential equation:

(7) $$J_{m}\theta_{m}^{\prime \prime }\left(t\right)+\gamma_{m}\theta_{m}^{\prime }\left(t\right)+K_{m}\theta_{m}\left(t\right)=T_{m}\left(t\right)$$

where $\theta_{m}$ is the modal angle, $J_{m}$ is the modal moment of inertia, $\gamma_{m}$ is the modal damping coefficient, $K_{m}$ is the modal torsional spring constant, and $T_{m}$ is the applied torque. The subscript *m* indicates the actuation (a) or detection (d) mode. The channel is actuated by a Lorentz force with an external magnetic field ***B*** and an AC current through the metal tracks on top of the channel. The actuation torque $T_{a}$ can be expressed as

(8) $$T_{a}\left(t\right)=L_{x}L_{y}Bi_{a}\cos\left(\omega_{a}t\right)$$

When the actuation is at resonance, the quality factor $Q_{a}$ and a −90° phase shift should be taken into account. Then, the modal angle can be expressed as

(9) $$\theta_{a}\left(t\right)=\frac{L_{x}L_{y}BQ_{a}}{K_{a}}i_{a}\cos\left(\omega_{a}t-\frac{\pi }{2}\right)$$

So the actuation angular velocity $\omega_{act}$ is then

(10) $$\omega_{act}\left(t\right)=\theta_{a}^{\prime }\left(t\right)=-\frac{L_{x}L_{y}BQ_{a}\omega_{a}}{K_{a}}i_{a}\cos\left(\omega_{a}t\right)$$

According to Equation (1), the Coriolis force is proportional to the cross-product of $\omega_{act}$ and $\Phi_{m}$ and only on the tube segment parallel to *x*-direction. One can then write

(11) $$T_{d}\left(t\right)=L_{y}F_{c}=-2L_{x}L_{y}\Phi_{m}\omega_{act}\left(t\right)=\frac{2L_{x}^{2}L_{y}^{2}BQ_{a}\omega_{a}}{K_{a}}\Phi_{m}i_{a}\cos\left(\omega_{a}t\right)$$

This torque excites the detection mode with its own differential equation (Equation (7)) and its own resonance frequency $\omega_{d}$. Two cases need to be discussed separately to solve this: $\omega_{a}\gg \omega_{d}$ and $\omega_{a}\ll \omega_{d}$. In the first case, where the actuation resonance frequency is much higher than the detection one, the detection mode is then actuated above its resonance frequency. This results in a 180° phase shift and a lowering of the amplitude by a factor of ${\left(\omega_{d}/\omega_{a}\right)}^{2}$. So the detection angle is written as

(12) $$\theta_{d}\left(t\right)={\left(\frac{\omega_{d}}{\omega_{a}}\right)}^{2}\;\cdot \;\frac{T_{d}\left(t\right)}{K_{d}}={\left(\frac{\omega_{d}}{\omega_{a}}\right)}^{2}\;\cdot \;\frac{2L_{x}^{2}L_{y}^{2}BQ_{a}\omega_{a}}{K_{a}K_{d}}\Phi_{m}i_{a}\cos\left(\omega_{a}t-\pi \right)$$

Therefore, the ratio between detection and actuation modal angle amplitude is

(13) $$\frac{\theta_{d}}{\theta_{a}}=\frac{2L_{x}L_{y}\omega_{d}^{2}}{K_{d}\omega_{a}}\Phi_{m}$$

In the second case where the actuation resonance frequency is much lower than the detection one, the detection mode is then excited quasi-statically, and can be written as

(14) $$\theta_{d}\left(t\right)=\frac{2L_{x}^{2}L_{y}^{2}BQ_{a}\omega_{a}}{K_{a}K_{d}}\Phi_{m}i_{a}\cos\left(\omega_{a}t\right)$$

and the ratio between detection and actuation modal angle amplitude is then

(15) $$\frac{\theta_{d}}{\theta_{a}}=\frac{2L_{x}L_{y}\omega_{a}}{K_{d}}\Phi_{m}$$

As will be explained in Section 2.4, in our case, the resonance frequency of the twist mode is higher than that of the swing mode. In this work, the twist mode is used for actuation and the swing mode is used for detection; therefore, Equation (13) applies. The modal angles can be expressed as

(16) $$\left\{\begin{array}{c} sin\left(\theta_{d}\right)=\frac{D_{2}}{L_{y}}\\ sin\left(\theta_{a}\right)=\frac{2(D_{1}-D_{2})}{L_{x}} \end{array}\right.$$

where $D_{1}$ and $D_{2}$ are the amplitudes of the displacement of points $P_{1}$ and $P_{2}$. The amplitude of the Coriolis force-induced movement is usually much smaller than the amplitude of actuation [24], so

(17) $$D_{1}-D_{2}\approx D_{1}$$

With small angles and by inserting Equation (17) into Equation (16), one can have

(18) $$\left\{\begin{array}{c} \theta_{d}=\frac{D_{2}}{L_{y}}\\ \theta_{a}=\frac{2D_{1}}{L_{x}} \end{array}\right.$$

Then, by substituting the modal angles $\theta_{d}$ and $\theta_{a}$ in Equation (13), one can derive

(19) $$\frac{D_{2}}{D_{1}}=\frac{4L_{y}^{2}\omega_{d}^{2}}{K_{d}\omega_{a}}\Phi_{m}$$

Hence, the mass flow can be estimated by measuring the displacement amplitude of points $P_{1}$ and $P_{2}$.

### 2.4. Sensor Design

**Figure 2:**
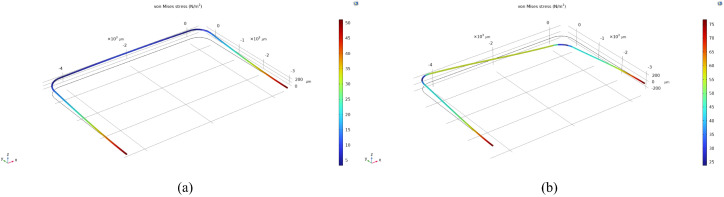
COMSOL Multiphysics^®^ simulation of the resonance modes of the tube. (**a**) Swing mode, resonance frequency 5095.5 Hz. (**b**) Twist mode, resonance frequency 10,754 Hz.

**Figure 3:**
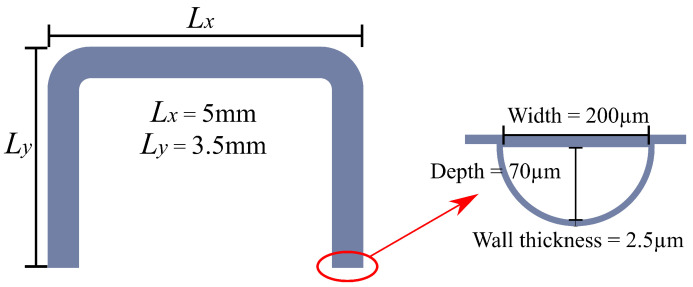
Top view of the designed tube structure of the sensor and the cross-section of the tube. Both ends of the tube are fixed and connected to the inlet/outlet.

According to the study presented by Haneveld et al. [15], the frequencies of different resonance modes should be sufficiently separated in order to increase the sensitivity and stability of the sensor. Simulations were performed with COMSOL Multiphysics^®^ 6.1 to determine the resonance frequencies, shown in Figure 2. Based on this, the sensor was designed with tube dimensions of 3.5 mm ($L_{y}$) and 5 mm ($L_{x}$). The cross-section of the tube is semi-circular due to the etching method and has a width of 200 μm and a depth of 70 μm, shown in Figure 3.

## 3. Fabrication Process

From a bare silicon wafer to a working Coriolis mass flow sensor device, the complete fabrication process is long and complex. So, it is divided into different parts and described with details and discussion of the key steps. The overall fabrication process can be presented as follows:

- Channel etching;
- Inlet/outlet etching;
- Channel wall formation;
- Integration of actuation/readout system;
- Channel release etching.

Table 1 lists the used materials and corresponding colors as used in the figures in this paper.

### 3.1. Channel Etching

**Figure 4:**
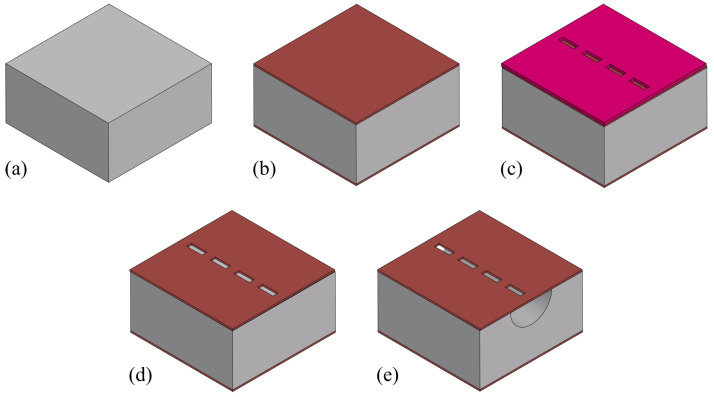
Fabrication steps to etch surface channels. (**a**) Wafer inspection. (**b**) SiRN deposition by LPCVD. (**c**) Photoresist spin coating, exposure, and development. (**d**) Pattern transfer to SiRN layer by directional plasma etching. (**e**) HNA etching to form the channel.

Figure 4 shows the steps for etching surface channels. The process starts with a double side polished silicon wafer (Figure 4a). First, a 1 μm thick SiRN layer is deposited using LPCVD (Figure 4b). Photoresist is then spin-coated onto the wafer, and exposed with a pattern containing arrays of 5μm×12μm slits, with a distance of 8 μm between each slit, and developed (Figure 4c). The slit width is determined by the layer thickness of channel wall to be deposited. The pitch is set in the range to prevent a scallop structure at the bottom of the channel, as the channel is created by connecting etched semi-spherical cavities. The pattern is transferred to the SiRN layer by directional plasma etching (Figure 4d). The silicon beneath is etched using an HNA solution (a mix of hydrofluoric acid, nitric acid, and acetic acid). This isotropic etching creates semi-spherical cavities under each slit. As the etching continues, these cavities connect to form a surface channel (Figure 4e).

**Figure 5:**
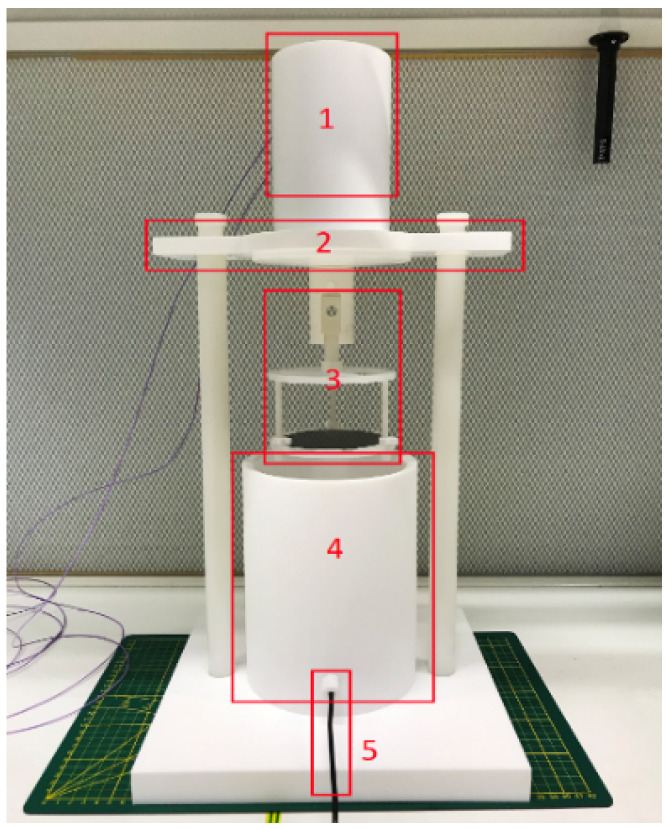
Dedicated reaction vessel for HNA etching. (**1**) Rotation motor. (**2**) Lid. (**3**) Wafer carrier. (**4**) Container. (**5**) Thermocouple.

**Figure 6:**
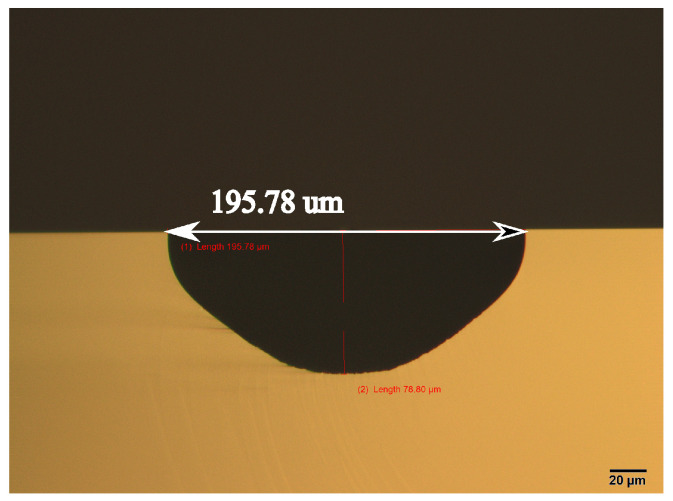
Optical microscope image of the cross-section of the etched dummy wafer.

From the literature, it is known that the etching profile, like the etch-rate, etched structure shape, etc., is highly dependent on the aperture [25]. The used composition here has a volumetric ratio of 50% HF solution, 69% HNO_3_ solution, and acetic acid in a ratio of 2:7:1. In a dedicated vessel, 1 L HNA solution is freshly prepared [26] for this process (see Figure 5). A dummy wafer is first etched to test. The wafer is mounted to the carrier and then submerged into the solution. Wafer rotation is added with a speed of 10 RPM to improve the etching uniformity [27,28]. The total etching time is 50 min, and the result is shown in Figure 6. It can be seen that the cross-section is more semi-elliptical than semi-circular. It has a width of 195 μm and depth of 79 μm, so the etching is not fully isotropic. Roughness can be clearly seen at the bottom of the channel. This is due to the sedimentation of the etching product, and it slows down or even prevents further reaction. This also explains the cross-sectional shape since the depth is expected to be half of the width.

**Figure 7:**
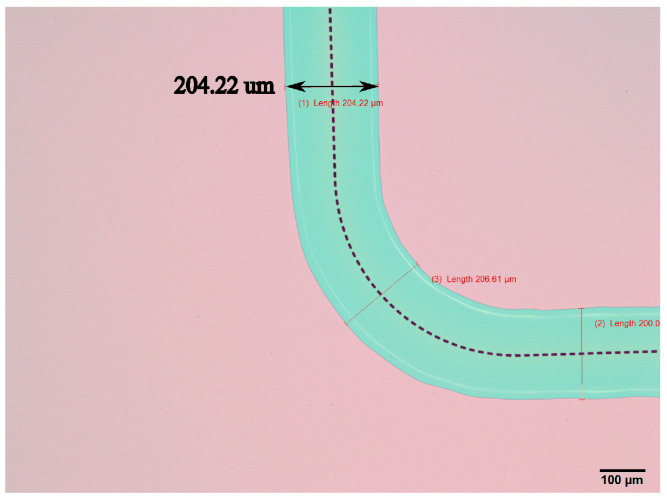
Top view of the etched channel by an optical microscope.

The device wafer is processed in the same way and etched for 53 min to reach 200 μm channel width. The etched channel is inspected from the top with an optical microscope to check the dimension thanks to the transparency of the SiRN thin film under white light. The result is shown in Figure 7. Alignment marks are designed in the pattern as well for subsequent steps. During SiRN etching, Kapton tape is applied to the alignment marks during the etching step to protect the silicon underneath the mark in later HNA etching.

### 3.2. Inlet/Outlet Etching

**Figure 8:**
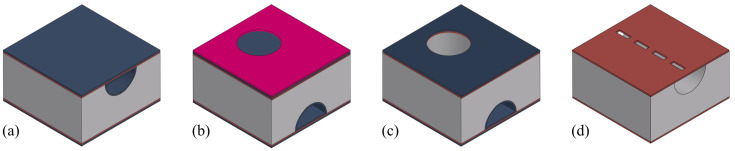
Fabrication steps to etch inlet and outlet. (**a**) TEOS deposition by LPCVD. (**b**) Photoresist spin coating, exposure, and development. (**c**) Pattern transfer to TEOS and SiRN, then Si DRIE. (**d**) Removal of TEOS layer by HF.

Once the channel is formed, inlets and outlets are etched from the back of the wafer. Figure 8 shows the steps. First, a 1 μm thick TEOS layer is deposited using LPCVD (Figure 8a). This TEOS layer acts as a hard mask and an etch-stop for the inlet/outlet etching. It needs to be thick enough because the silicon wafer will be etched all the way through in the next step. Next, photoresist is spin-coated onto the back of the wafer (Figure 8b). The pattern for the inlets and outlets is aligned with the channel using backside alignment and then exposed and developed. The photoresist used here should be thick enough to ensure the pattern transfer to the TEOS and SiRN layers. Deep reactive ion etching (DRIE, Bosch process) is then used to etch the inlet/outlet down to the TEOS channel wall (Figure 8c). The silicon is etched to a depth of about 450 μm. The etching progress is checked by illuminating the backside of the wafer. If the light is visible from the front through the inlet/outlet openings, it means the etching has reached the TEOS channel wall. After the Bosch process, O_2_ plasma is used to clean the wafer and remove fluorocarbon residues and remaining photoresist. Finally, the TEOS layer is removed by HF to connect the channel to the inlet/outlet (Figure 8d).

### 3.3. Channel Wall Formation

Due to the dimension of the designed slit pattern (5μm×12μm), a single-layer SiRN deposition is not sufficient to close the apertures. Therefore, a multiple-layer stack is needed. The inner wall material should be SiRN due to its chemical resistance. As for the outer layer, TEOS is used for its sufficient protection during the subsequent release etching step and relatively low intrinsic stress (compared to thermal oxide).

**Figure 9:**
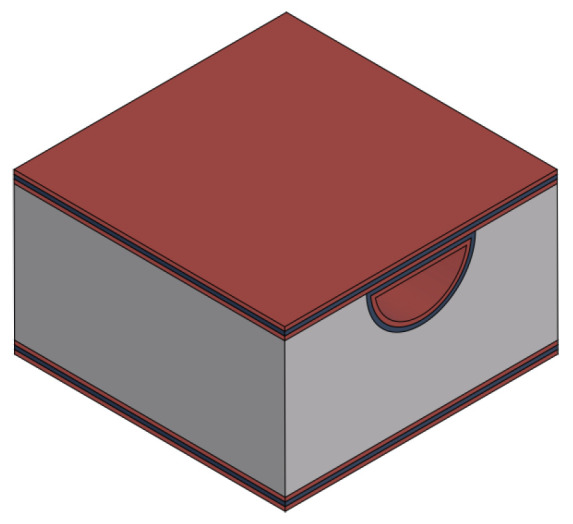
Fabrication step to form channel wall and close the slits by TEOS and SiRN deposition.

To completely close the slits, a minimum layer thickness of 2.5 μm is needed. Considering that SiRN is also etched during channel etching, although very slowly, over-deposition is certainly desired. So, a 1.1 μm thick TEOS layer is deposited, followed by a 1.6 μm thick SiRN layer deposition (Figure 9). To verify that the slits are completely closed, water droplets are spilled on the wafer. After drying, the wafer is inspected with an optical microscope. If the slits are completely closed, no liquid residue should be observed inside the channels.

After this step, the channel is now connected to the outer environment only through the inlet and outlet. Any liquid residue in the channel stays and is hard to remove. Therefore, it is suggested to avoid any wet processes. If needed, a foil should be applied on the backside of wafer to prevent any wet chemicals or water entering through the inlet/outlet.

### 3.4. Integration of Actuation/Readout System

**Figure 10:**
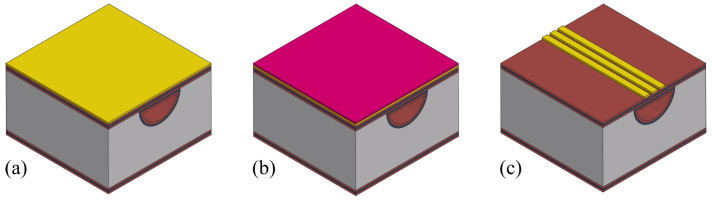
Fabrication steps to create electrodes for the actuation/readout system. (**a**) Metal sputtering. (**b**) Photoresist spin coating, exposure, and development. (**c**) Pattern transfer to metal layer by ion beam etching.

There are numerous actuation and readout methods, and the required structures are also different [16,17]. Lorentz force actuation and vibrometer detection are chosen in this work for the ease of designing the system. Figure 10 illustrates the fabrication steps to create the electrodes. It starts with metal sputtering. Depending on the application, different metals can be chosen. In our application, durability and conductivity are important features; therefore, platinum and gold are usually used, additionally with a thin tantalum layer underneath for good adhesion. So, a stack of 10 nm tantalum, 20 nm platinum, and 200 nm gold layers (from bottom up) is sputtered on the substrate consecutively without breaking vacuum (Figure 10a). Photoresist is spin-coated (Figure 10b), exposed, and developed. To etch the metal layers, ion beam etching (IBE) is used. To monitor the process for end-point detection, secondary ion mass spectrometry (SIMS), which is integrated into the etching system, is used. After that, O_2_ plasma is used to clean the wafer and remove the remaining photoresist (Figure 10c).

### 3.5. Channel Release Etching

**Figure 11:**
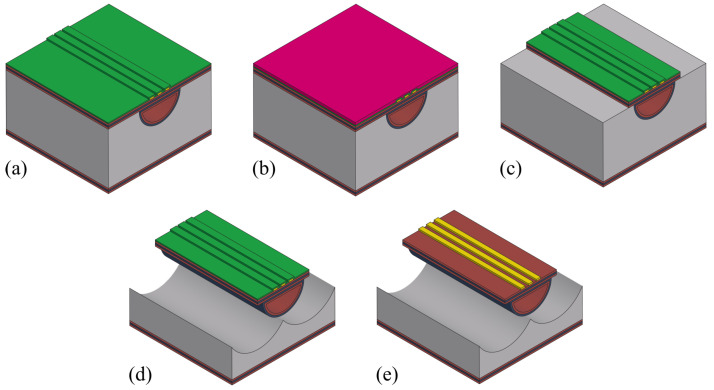
Fabrication steps to release the channel. (**a**) Aluminum oxide deposition by E-beam evaporation. (**b**) Photoresist spin coating, exposure, and development. (**c**) Patterning of aluminum oxide, SiRN, and TEOS layers. (**d**) Bulk silicon etching by SF_6_ plasma. (**e**) Removal of remaining aluminum oxide layer.

To allow the vibration, the channel needs to be released from the substrate. This is performed by etching away the bulk silicon around the channel. Figure 11 shows the fabrication steps. Considering the volume of silicon material to be etched, a durable mask layer is necessary to protect fabricated structures (metal traces, channel, etc.). Aluminum oxide is used as hard mask material because of its significantly low etch rate during the plasma etching of silicon. The aluminum oxide layer is deposited by electron beam evaporation and covers the metal traces on the wafer (Figure 11a). Photoresist is then spin-coated (Figure 11b), exposed, and developed. Boron trichloride (BCl_3_) and hydrogen bromide (HBr) gases are used to etch aluminum oxide for patterning. The SiRN and TEOS layers underneath are then etched through (Figure 11c) using SF_6_ so that the silicon underneath is exposed.

SF_6_ plasma is used for bulk silicon etching. During the process, silicon around the channel is removed (Figure 11d). Therefore, heat dissipation becomes critical, and the channel wall material can become burned by the plasma. From previous experiences with SCT, alternating etching and waiting can help to solve the issue, i.e., etch for a certain duration and then stop the plasma and wait for cooldown. After this step, the remaining aluminum oxide layer is stripped again with BCl_3_ and HBr gases to reveal the electrodes (Figure 11e).

### 3.6. Result

**Figure 12:**
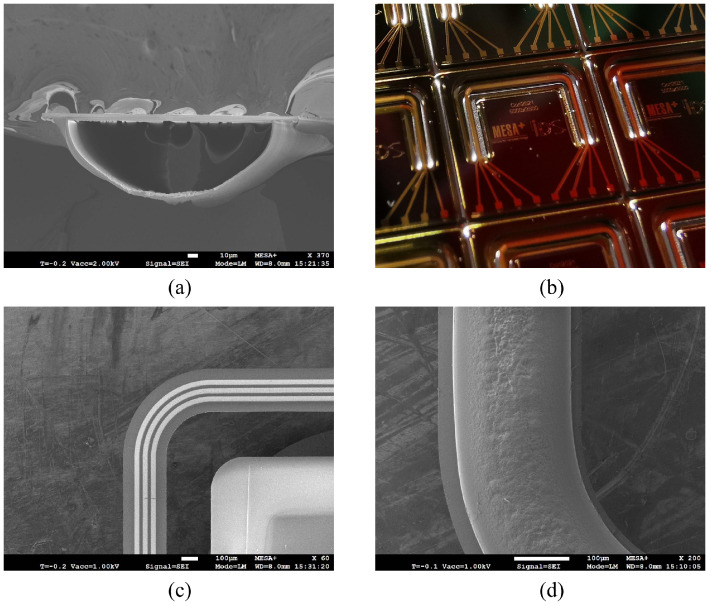
Pictures of fabrication results. (**a**) SEM image of the channel cross-section (© 2022 IEEE). (**b**) Photograph of a fabricated sensor chip just before the release from the wafer (© 2022 IEEE). (**c**) SEM image of the channel from the top view. (**d**) SEM image of the channel from the bottom view.

Figure 12 shows SEM images of the fabricated channel after being released from the substrate and a photograph of the sensor chips just before being released from the wafer. The channel cross-section is semi-elliptical, and has a width and depth of 200 μm and 72 μm (Figure 12a). The bulk silicon around the channel is fully removed, and the channel is free-suspended; only the ends are still anchored in the substrate and connected to the inlet/outlet (Figure 12b). Figure 12c shows the SEM image of the left corner area of the channel from a top view. The metal tracks are clearly visible. Figure 12d shows the rough surface at the bottom of the channel. This roughness is originally from the channel etching step in silicon, and now in the channel wall layer due to conformal deposition.

## 4. Measurements

### 4.1. Experimental Setup

**Figure 13:**
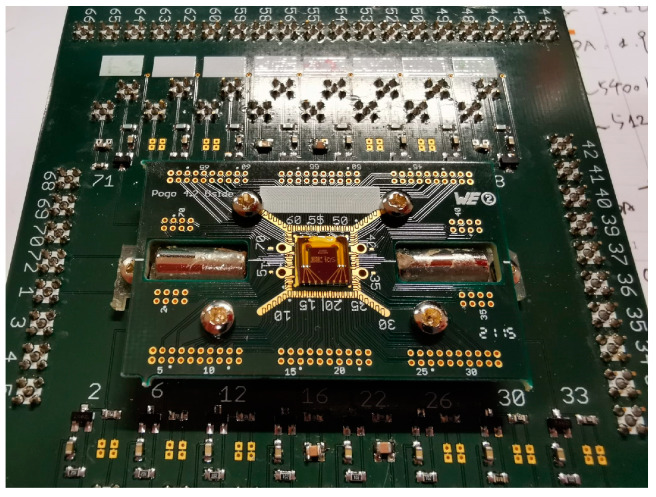
Photograph of a fabricated sensor chip after mounting to the circuitry. The small PCB is connected to the large board through pins. Permanent magnets are on the sides of the sensor chip. The block for fluid inlet/outlet is beneath the PCB.

After the fabrication of a Coriolis mass flow sensor device, experimental measurements are carried out for demonstration. The sensor chip is first glued on a dedicated printed circuit board (PCB) with the inlet/outlet aligned to the holes on the PCB. The electrodes on chip are wire-bonded to the PCB. The PCB is then mounted and fixed on an electrical board with two permanent magnets for Lorentz actuation, pins that can connect to a power supply and a block that provides a fluid inlet/outlet. Figure 13 shows a photograph of a sensor chip after mounting to the circuitry. The board is then fixed on an active vibration isolation optical table for the measurement.

**Figure 14:**
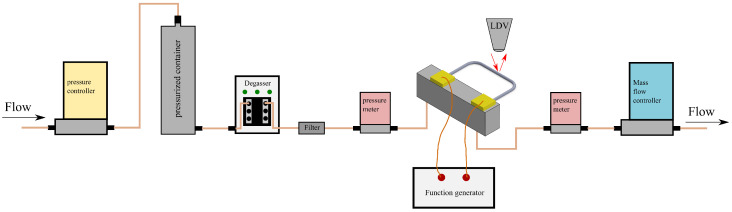
Schematic of the measurement setup.

Figure 14 illustrates the schematic of the measurement setup for liquid. A nitrogen gas line is connected to a pressure controller to set the input pressure of liquid in a pressurized container. A degasser is connected to prevent any bubbles in the liquid. A filter is connected after the degasser to remove particles in the liquid. A Bronkhorst B.V. IQ+FLOW pressure sensor, which has an accuracy of 0.025 bar, is connected before the chip to read the actual input pressure of the liquid. The chip is connected to a sine wave generator for actuation. The movement of the sensor tube is then detected by a Laser Doppler Vibrometer (LDV), which is part of a Polytec MSA-600 microsystem analyzer. Another pressure sensor is connected to the chip outlet to read the output pressure. At the end of the line, a mass flow controller is connected to control the mass flow rate. For gas measurement, the pressurized container and degasser can be removed.

### 4.2. Measurement Method

The channel is actuated with 0.5 V input AC voltage to generate the current along the metal track so it vibrates. The resistance of the metal track is measured to be approximately 120 Ω. Thermal expansion may occur due to Joule heating, which changes the geometry; thus, the mechanical properties of the structure can change and affect the resonance frequency. Therefore, for measurements that require input actuation signal, the input signal should be applied, waiting for a certain period until the temperature of the channel is stable.

The interested parameters to be measured are the pressure drop of the fluid along the channel, the tube resonance frequencies, and the displacement of point P_1_ and P_2_ (shown in Figure 1) under different mass flow rates.

## 5. Result and Discussion

### 5.1. Pressure Drop Measurement

**Figure 15:**
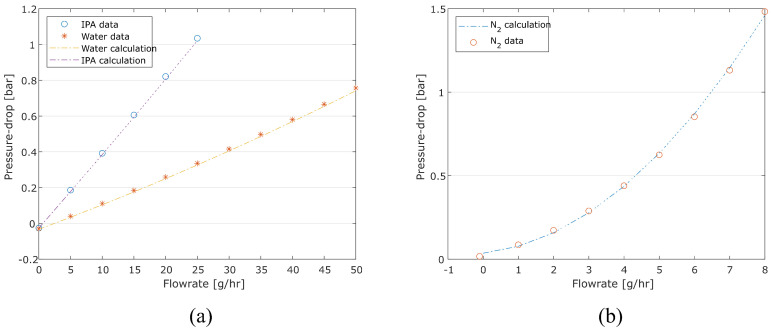
Measured pressure drop over the sensor tube with an input pressure of 4 bar as a function of mass flow rate for (**a**) water and isopropanol (IPA) (© 2022 IEEE). (**b**) nitrogen.

Pressure drop measurements are taken to identify the flow range. This is performed by reading two pressure sensors before and after the chip and calculating the difference. The design of the sensor is based on a maximum pressure drop of 1 bar. Figure 15 shows the plots of the measured pressure drop of water, IPA, and nitrogen over the sensor. As introduced in Equation (4), the pressure drop should be proportional to the mass flow rate.

The data points are fitted with a polynomial of degree two. According to the fit, it can be calculated by Equation (4) that the cross-section of the channel is equivalent to a circle with a diameter of approximately 100 μm for modeling the pressure drop. The value of the loss coefficient is also calculated and shown in Table 2.

It can be seen that the value of the loss coefficients of water and IPA are significantly lower than the one of nitrogen. For measurements of liquid, the relation between the pressure drop and the mass flow rate is almost linear, which means that the pressure drop due to the bends and junctions of the channel is negligible compared to that over the straight sections. As for gas measurement, it appears that there is a much higher pressure drop at the bends and junctions of the channel, which is not negligible, and the total pressure drop is dominated by this term.

For water measurement, a maximum flow range (50 $g\;h^{-1}$) of the mass flow controller is reached, while the pressure drop is only 0.747 bar. The estimated Reynolds numbers of nitrogen, water, and IPA at the designed maximum flow rates of the sensor (6 $g\;h^{-1}$ for nitrogen, 50 $g\;h^{-1}$ for water, and 25 $g\;h^{-1}$ for IPA) are 1205, 177, and 43, respectively.

### 5.2. Resonance Frequency Measurement

**Figure 16:**
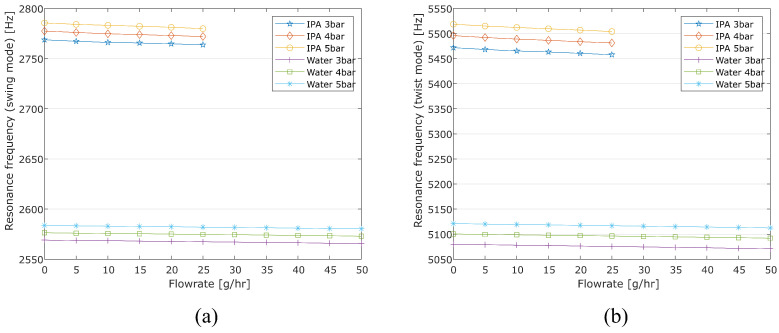
Measured resonance frequency of the sensor tube under different input pressure as a function of the mass flow rate of water and IPA. (**a**) Swing mode. (**b**) Twist mode (© 2022 IEEE).

The resonance frequencies need to be measured for density and mass flow calculations. Their relationship with the input pressure and flow rate is of interest. Figure 16 shows the measured resonance frequencies for both the actuation (twist mode) and detection (swing mode) of water and IPA under different input pressures and mass flow rates. For the same mass flow rate, the resonance frequency increases with the input pressure. This is because the tube deforms under pressure [18]. The resonance frequency is also affected by the mass flow rate. This happens because a higher mass flow rate causes a larger pressure drop (shown in Figure 15) across the sensor tube. This leads to a bigger pressure gradient along the tube, affecting the symmetry of the structure. Additionally, different fluids can lead to different resonance frequencies because the total mass of the fluid inside the tube changes. This feature can then be used to measure fluid density as discussed above.

### 5.3. Density Measurement

**Figure 17:**
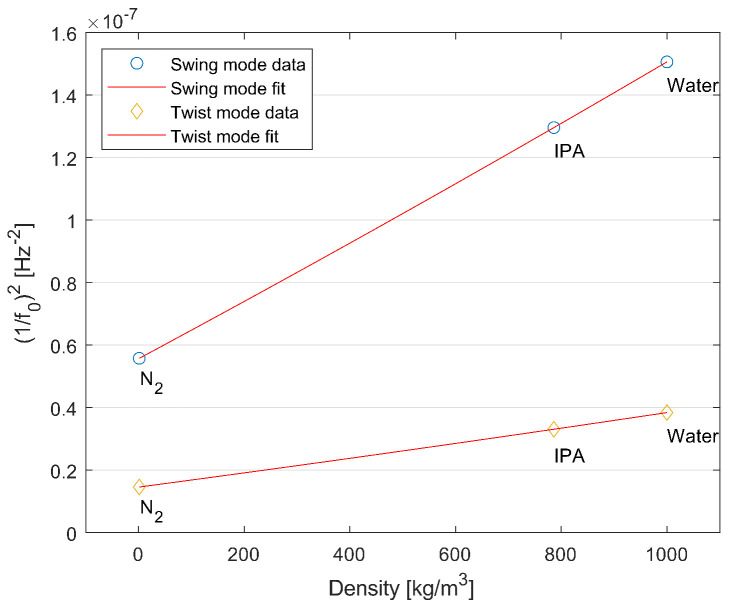
Measured resonance frequency of the sensor tube as a function of fluid media density at a constant pressure of 4 bar.

Density measurements are taken by measuring the resonance frequency of the tube filled with nitrogen, IPA, and water. The input pressure is set to 4 bar. Both twist and swing mode frequencies are recorded and plotted. Figure 17 shows the result. According to Equation (6), the relation between the fluid density and the corresponding resonance frequencies ${\left(1/f_{0}\right)}^{2}$ should be linear. The fitted straight lines are then a demonstration of a density sensor.

### 5.4. Mass Flow Measurement

In this experiment, the tube is actuated in twist mode, and the flow rate is detected in swing mode. From the resonance frequency measurement, one can know that the twist mode of the tube has a higher frequency. Therefore, for mass flow measurement, Equation (19) applies. Nitrogen, water, and IPA flows are measured. It is important to notice that when measuring different fluids (of different density) and comparing the results, the term $\omega_{a}/\omega_{d}^{2}$ should be multiplied with the displacement ratio to compensate for the change in resonance frequency, so Equation (19) can be written as

(20) $$\frac{D_{2}\omega_{a}}{D_{1}\omega_{d}^{2}}=\frac{4L_{y}^{2}}{K_{d}}\Phi_{m}$$

and the term on the left of the equation can be called the compensated displacement ratio.

**Figure 18:**
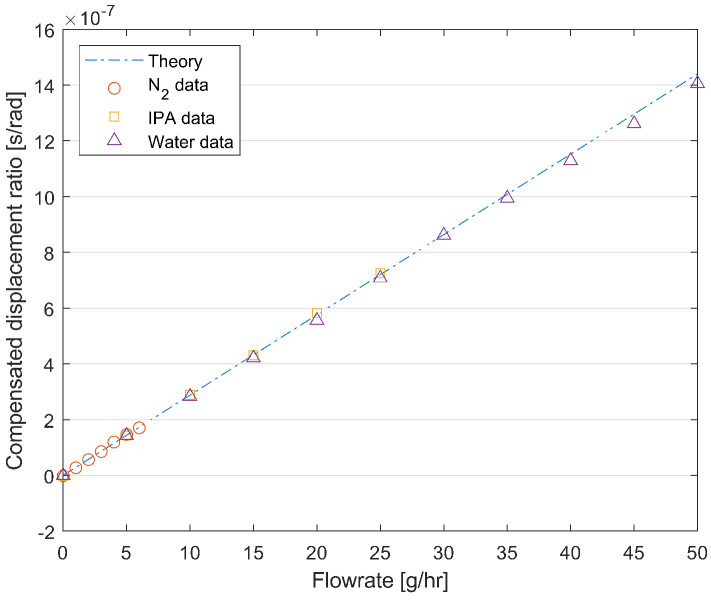
Measured compensated displacement ratio (Equation (20)) between point P_2_ and P_1_ as a function of the mass flow rate for nitrogen, water, and IPA with an input pressure of 4 bar and the modeled behavior (dashed line).

Figure 18 shows the measured ratio of the displacement amplitude at the center point P_2_ and the corner point P_1_ (shown in Figure 1) multiplied by the compensation factor mentioned above as a function of the mass flow rate of nitrogen, water, and IPA, with an input pressure of 4 bar. It shows a linear behavior that matches the theoretical calculation. Therefore, a true mass flow measurement is demonstrated.

### 5.5. Stability Measurement

**Figure 19:**
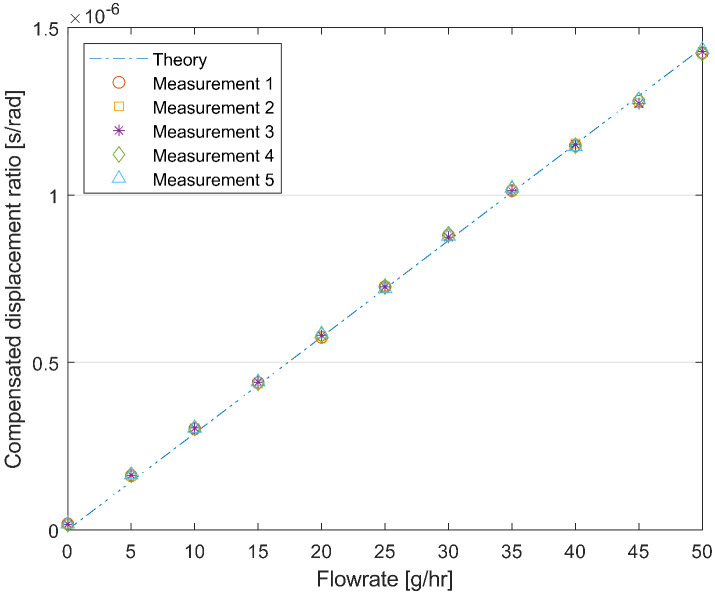
Repeatedly measured compensated displacement ratio (Equation (20)) between point P_2_ and P_1_ as a function of the mass flow rate for water with an input pressure of 4 bar.

Stability measurement of the device is taken by measuring the water flow repeatedly (input pressure 5 bar, flow range 0–50 $g\;h^{-1}$). The result is plotted in Figure 19. The measurements are taken starting from 0 to 50 $g\;h^{-1}$ with steps of 5 $g\;h^{-1}$, then reducing the flow rate from 50 $g\;h^{-1}$ to 0, again with 5 $g\;h^{-1}$ steps. The cycle of measurement is repeated five times. According to the calculation, the relative standard deviation (RSD) of each measured points is no more than 1%, excluding the 0 $g\;h^{-1}$ flow rate point.

## 6. Conclusions

This paper presents a free-suspended microfluidic tube with a semi-elliptical cross-section (200 μm wide, 70 μm deep) and a tube wall thickness of approximately 2.5 μm, created using a new fabrication process based on existing SCT and HNA etching. A Coriolis mass flow and density sensor is fabricated with this process and demonstrated. The large cross-sectional area leads to a significant improvement in the flow range (from 0 up to 50 $g\;h^{-1}$ for water and up to 6 $g\;h^{-1}$ for nitrogen gas) compared to devices fabricated with standard SCT [15]. Although the non-circular cross-sectional area affects the performance of the sensor due to the input pressure of the fluid, the effect is limited.

Future work will be focused on the optimization of both the fabrication process to realize a pressure-independent structure and the design of sensors for better performance. Different actuation and readout methods can be applied by integrating the system just like in standard SCT. Moreover, other fluid sensors/devices can also be integrated. For instance, by integrating pressure sensors on the chip, the pressure drop along the tube can be measured more accurately.

## Figures and Tables

**Table 1:** Legend for the materials used in the fabrication process.

Material	Name and Abbreviations
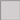	Silicon (Si)
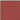	Silicon-rich silicon nitride (SiRN)
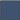	Tetraethyl orthosilicate (TEOS)
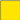	Metal stack of Ta, Pt and Au (Metal)
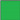	Aluminum oxide (Al_2_O_3_)
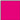	Photoresist (PR)

**Table 2:** Value of the loss coefficient κ in Equation (4) for different fluids.

Fluid	Unit	Value
Water	[-]	5.63 × 10^−18^
IPA	[-]	4.64 × 10^−18^
N_2_	[-]	2.58 × 10^−14^

## Data Availability

The original contributions presented in the study are included in the article, further inquiries can be directed to the corresponding author/s.
